# Carbon Ion Irradiation Enhances the Anti-tumor Efficiency in Tongue Squamous Cell Carcinoma via Modulating the FAK Signaling

**DOI:** 10.3389/fpubh.2021.631118

**Published:** 2021-02-03

**Authors:** Qingzong Si, Qian Ye, Zhitong Bing, Ruihong Fan, Xiaoli Hu, Bin Liu, Jizeng Wang, Yang Liu, Xiaoli An

**Affiliations:** ^1^School of Stomatology, Lanzhou University, Lanzhou, China; ^2^Institute of Modern Physics, Chinese Academy of Sciences, Lanzhou, China; ^3^Institute of Solid Mechanics, School of Civil Engineering and Mechanics, Lanzhou University, Lanzhou, China

**Keywords:** carbon ion irradiation, FAK, metastatic potential, tongue squamous cell carcinoma, cell growth

## Abstract

Oral cancer is a very aggressive disease with high rates of recurrence and metastasis. This study aimed at addressing how efficiently tongue cancer is suppressed after carbon ion irradiation. Here, the close relationship between upregulated expression of focal adhesion kinase (FAK) and high metastatic status in tongue squamous cell carcinoma patients was validated using bioinformatics and immunohistochemical analyses. Our data indicated that FAK suppression significantly enhanced the killing effect induced by irradiation in the tongue cancer cell line CAL27, as evidenced by increased apoptotic induction and reduced colony formation. More importantly, in FAK-deficient cells, carbon ion irradiation was shown to remarkably inhibit migration and invasion by delaying wound healing and slowing down motility. Further studies revealed that irradiation exposure caused disorganization of the actin cytoskeleton and reduced cell adhesive energy in FAK-deficient cells. Moreover, carbon ion treatment, in combination with FAK silencing, markedly blocked the phosphorylation levels of FAK, and paxillin, which partly contributed to the reduced motility of tongue squamous cell carcinoma CAL27 cells. Collectively, these results suggest that the prominent obstructing role of carbon ion irradiation in the growth inhibition and metastatic behavior of tumors, including attenuation of cell adhesiveness, motility, and invasiveness, could be distinctly modulated by FAK-mediated downstream pathways.

## Introduction

Oral squamous cell carcinoma (OSCC) is the most lethal head and neck squamous cell carcinoma, with an increasing incidence among younger subjects (1, 2). Over the past decades, the prognosis of OSCC patients has remained dismally poor despite remarkable improvements in surgery, chemotherapy, and radiotherapy (3–5). A retrospective review of clinical outcomes showed that the 5-year survival rate of patients with OSCC who underwent postoperative radiation therapy (PORT) using cobalt 60 photons was prominently lower than those who did not undergo PORT (6). Moreover, approximately one-third of patients with OSCC experience locoregional recurrence or distant metastases after multimodality management, including PORT (7). Hence, the development of more effective radiation treatment for OSCC therapy is imperative.

Growing evidence indicates that radiotherapy with heavy ions is advantageous in clinical trials compared to conventional irradiation with photons, such as γ-rays or X-rays, owing to the unique characteristics of improved dose deposition and higher relative biological effectiveness (8, 9). Carbon ion irradiation is more efficient in inducing cell killing than X-ray irradiation (10). Multiple lines of evidence from *vitro* and *in vivo* studies have shown that conventional radiotherapy can enhance the formation of metastasizing cells (11–13). In contrast, heavy ion irradiation has mostly been found to suppress the migratory and invasive potential of cancer cells (14–17). Our previous data also demonstrated that cell motility was more suppressed after carbon ion irradiation than after X-ray irradiation in glioma cells (18), lung cancer cells (19), and tongue squamous cell carcinoma (TSCC) (20).

Focal adhesion kinase (FAK) is frequently overexpressed in various tumors and is a crucial signaling component that is activated by numerous stimuli and functions as a biosensor or integrator for regulating cell motility, adhesion, and growth (21). FAK amplification in OSCC was reported to correlate with lymph node metastasis (22). Knockdown of FAK has been found to inhibit the survival, invasion, and metastasis of oral cancer (2, 23). Hence, the control of growth and metastatic processes will lead to promising therapies for the clinical treatment of OSCC by targeting FAK.

This study aimed to unravel the influence and possible mechanisms of carbon ion irradiation on metastatic potential in TSCC, one of the most common oral cancers (24). Moreover, we explored the contribution of FAK signaling as a modulator of behavior in cancer cells receiving carbon ion irradiation.

## Materials and Methods

### Clinical Data Collection and Processing

The genomic and clinical data of squamous cell carcinoma of the patients with oral cancer were extracted from head and neck squamous cell carcinoma data in The Cancer Genome Atlas (TCGA) database (http://xena.ucsc.edu/). The TCGA dataset included 124 primary tumors samples and 13 normal samples. To analyze the relationship between FAK and metastasis in oral TSCC, we extracted stage I (*n* = 9) and IV (*n* = 47) cancer samples from clinical data. The area of the receiver operating characteristic curve (AUC) represents the performance of each gene.

### Cell Culture and Treatment

The human TSCC cell line (CAL27) was purchased from BeNa Culture Collection (BNCC, Beijing, China). Lentiviral particles designed to silence human FAK (5-GATAGTGGACAGTCACAAA-3) and control lentiviral vectors were produced by Shanghai GeneChem Co. Ltd., China.

Carbon ion irradiation was conducted at the Heavy Ion Research Facility, Lanzhou of the Institute of Modern Physics, Chinese Academy of Sciences, using an 80 MeV/u carbon ion beam, with an LET 50 Kev/μm (18, 25).

### Colony Formation Assay

We measured the colony forming ability of the irradiated CAL27 cells with or without FAK modification. The fixed colonies with chilled methanol were stained with 0.4% crystal violet (Sigma-Aldrich). Colonies of >50 cells were used to analyze the cloning efficiency.

### Apoptosis Analysis by Flow Cytometry

Apoptosis was detected in irradiated CAL27 cells with or without FAK downregulation using a commercial kit (BD Biosciences, San Jose, CA, USA) according to the manufacturer's protocol. The apoptotic population was measured using a Flowsight imaging flow cytometer (Amnis/Merck Millipore, Darmstadt, Germany).

### Wound Healing Assay

We determined the migratory ability via wound assays using IBIDI culture-inserts (ibidi, Martinsired, Germany). The cells at the logarithmic growth stage were incubated at 37°C in a humidified atmosphere containing 5% CO_2_. When the cells were adherent to the wall in a single layer, a circular wound was scratched using a sterile 200 μL pipette tip. Cells migrated into the wounded area, and photographs were captured immediately (0 h) and at 6, 12, 24, and 48 h using an optical microscope. The wound area and migration velocity were analyzed using ImageJ software (National Institute of Health, USA).

### Transwell Assay

Cell invasion was assessed using BD Matrigel invasion chambers (BD Biosciences). Following FAK silencing and/or irradiation, CAL27 cells were seeded into the upper culture compartments supplemented with serum-free medium. The lower culture compartments were filled with DMEM containing 10% fetal bovine serum. Cells that invaded through the pores were fixed and stained with crystal violet after 24 h incubation.

### AFM for Imaging and Mechanical Measurements

Single-cell topographical and mechanical characteristics of living CAL27 cells were measured by a AFM nano-indentation method (JPK Instruments AG, Germany), as described previously (26, 27). Adhesion energy in the cytoplasmic regions was obtained using the JPK data processing software (Version spm-4.2.50, JPK, Germany) (28, 29).

### Immunofluorescence/Immunohistochemical Staining Assay

For F-actin staining, the fixed cells were stained with fluorescein isothiocyanate-phalloidin (Sigma-Aldrich, St. Louis, MO). The slides were mounted in VECTASHIELD with DAPI (Vector Laboratories, Burlingame, CA) and were viewed using a confocal microscope. The cytoskeleton obtained by immunofluorescence staining can be extracted using ImageJ software (NIH, USA), and the cytoskeleton structure was lined and analyzed. The cytoskeleton was marked with different colors according to the complexity of its connections, and the complexity of the cytoskeleton can be determined according to the distribution of colors.

Tissue microarray chips containing 20 tissues of TSCC were obtained from Shanghai Biochip Co., Ltd (HOraC060PG01 and Horac080PG01, and the ID of ethics approval was T20-0361). FAK expression was detected in cancer and juxtacancerous tissue (JCT) using immunohistochemical staining.

### Western Blot Analysis

Protein samples were extracted from cells in RIPA buffer (Solarbio, Beijing, China) and analyzed in cells 24 h after irradiation with or without FAK knockdown. Total protein samples were blotted with the following antibodies: anti-FAK, anti-phospho-FAK (Y397), anti-phospho-paxillin (Y118), and anti-β-actin (GeneTex, Irvine, CA).

### Statistical Analysis

Three independent experiments were carried out to obtain the quantitative data. Statistical analysis was performed using SPSS 16.0 (SPSS Inc., Chicago, IL, USA). Comparisons between two groups were performed with one-way analysis of variance (ANOVA) followed by LSD *post hoc* test. A P-value of <0.05 was considered statistically significant.

## Result

### Screening and Validation of TSCC Biomarkers Based on Bioinformatics Analysis

**Figure 1:**
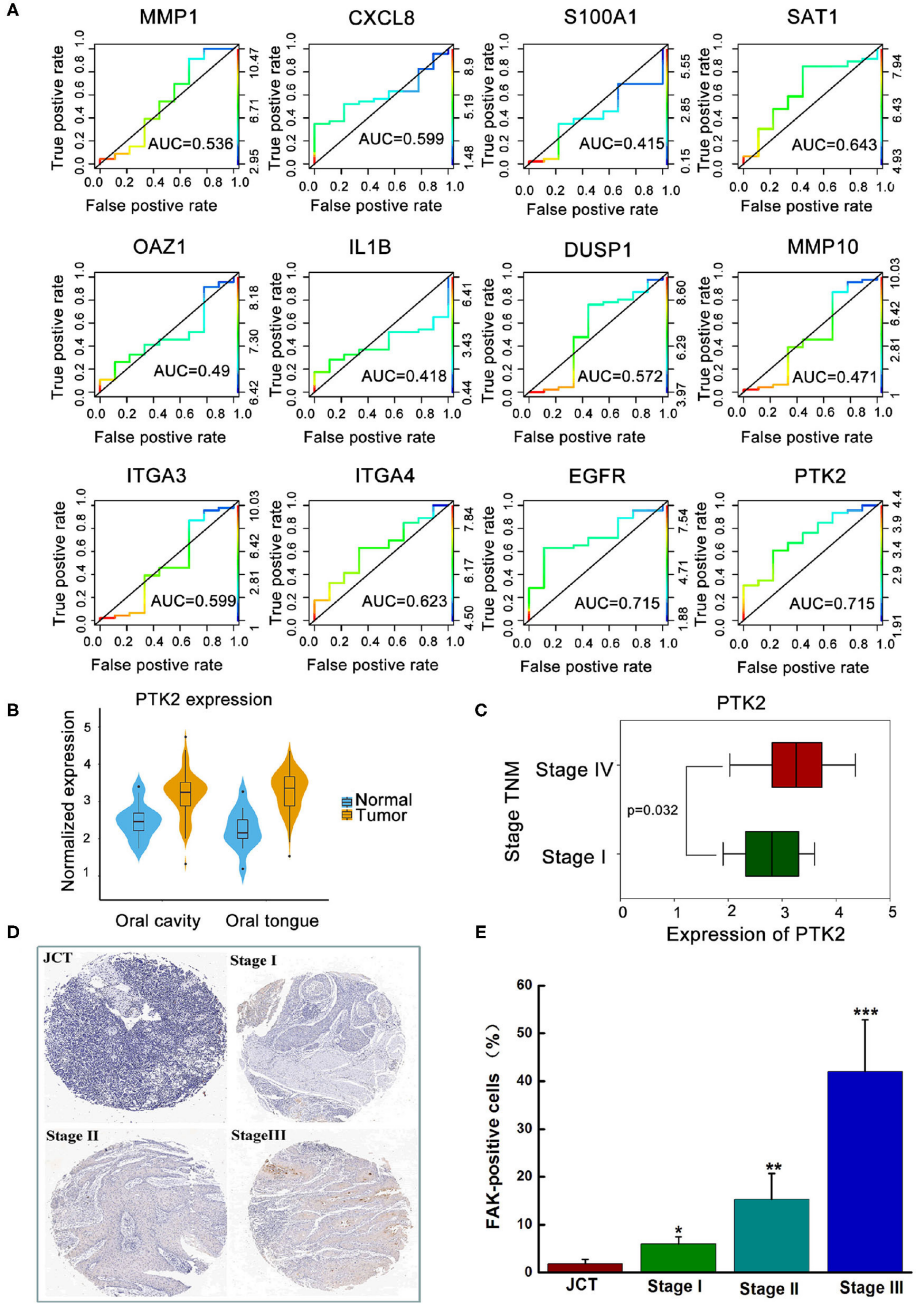
The clinical analysis between the expression of FAK and oral cancer progression. **(A)** Exploration of key genes involved in primary (N0) and metastatic (≥N1) tumors via the ROC curve test. **(B)** Higher expression of PTK2/FAK in the oral cavity or oral tongue cancer. **(C)** Difference between PTK2/FAK expression in stages I and IV tongue squamous cell carcinoma. **(D)** Detection of the expression of FAK in the tissue chip. **(E)** Quantitative analysis of FAK positive cell proportion. *P < 0.05, **P < 0.01, and ***P < 0.001 vs. the juxtacancerous tissue (JCT) group.

Based on previous literature, 12 transcriptomic biomarkers that are OSCC diagnostic biomarkers were screened in our study (30). As displayed in Figure 1A, the diagnosis and classification of primary (N0) and metastatic (≥N1) tumors using receiver operating characteristic curves were tested. The results showed that MMP-1, DUSP1, and ITGA3 were downregulated in OSCC, with AUC values of 0.536, 0.527, and 0.599, respectively. Conversely, SATA1, CXCL8, ITGA4, EGFR, and PTK2/FAK were upregulated in OSCC. More importantly, EGFR and PTK2/FAK had the highest AUC value (0.715) among all genes, indicating that EGFR and PTK2/FAK could be suitable biomarker candidates associated with cancer progression.

Considering the different locations of the primary oral tumor, Figure 1B shows a higher PTK2/FAK expression in oral cavity or oral tongue cancer. Boxplot analysis of stage I and IV samples indicated that PTK2/FAK was more prominently upregulated in stage IV than in stage I samples in the TSCC patients (*P* = 0.032, Figure 1C). Moreover, there was increased expression of FAK protein in the late stage of the tumor compared to the adjacent normal tissue in the TSCC patients using the tissue chip technique (Figures 1D,E). Taken together, these findings imply that PTK2/FAK is highly associated with the metastasis progression of TSCC patients.

### Target Effects of FAK Signaling in TSCC Cells Exposed to Carbon Ion Irradiation

**Figure 2:**
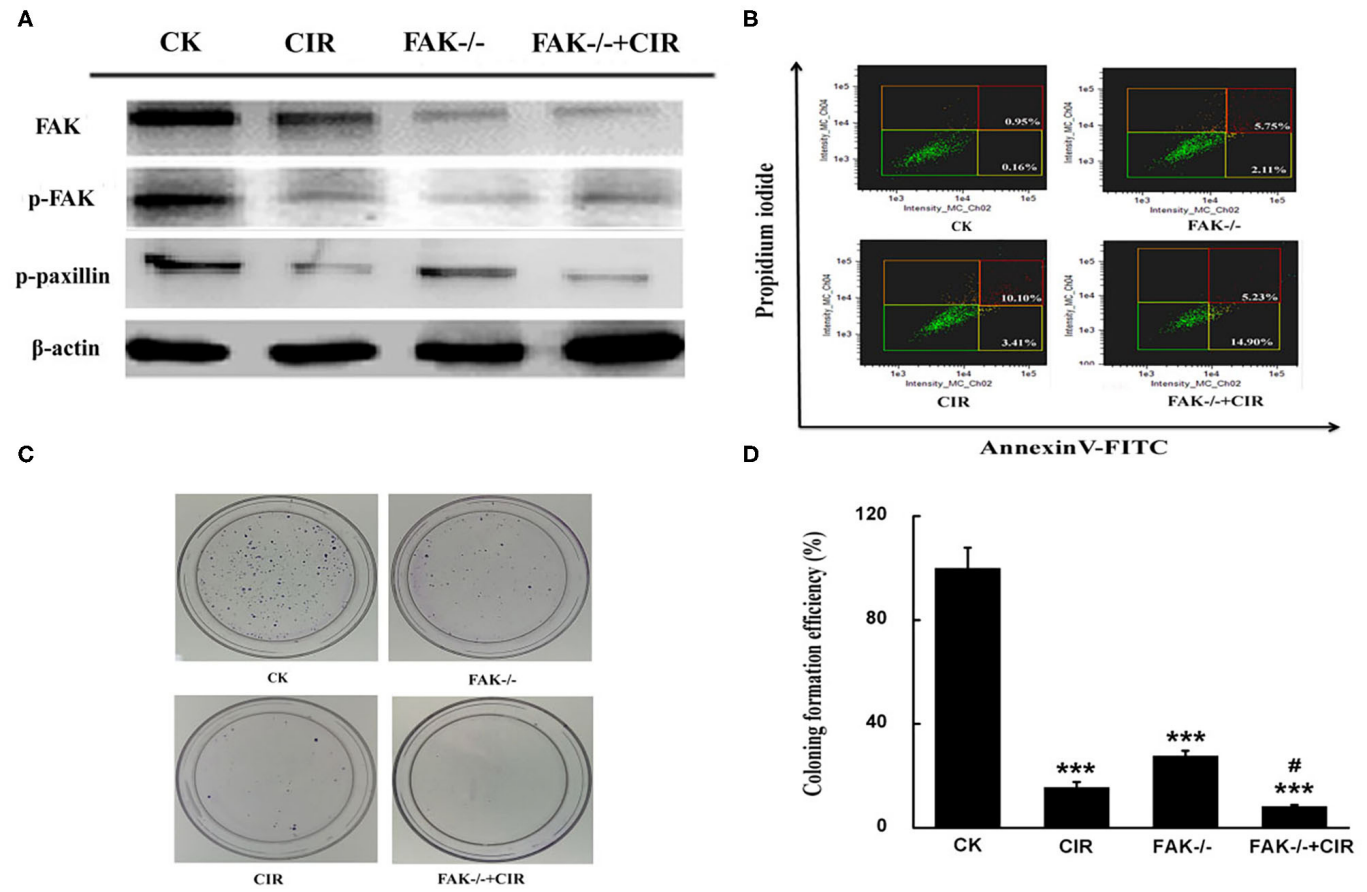
FAK depression is effective at inhibiting the growth of CAL27 cells. **(A)** Detection of FAK, FAK phosphorylation at Y397, and paxillin phosphorylation at Y118 expression. **(B)** Determination of the proportion of apoptotic cells by flow cytometry. **(C,D)** Representative images of the colony formation and quantitative evaluation of cell proliferation ability using clonogenic survival assays. The means ± SEM (*N* = 3) were calculated for each value. ***P < 0.001 vs. the control group. ^#^*P* < 0.05 vs. the irradiation group.

To test the contribution of FAK signaling in tumor inhibition during carbon ion radiation therapy, we knocked down FAK expression in CAL27 cells using lentivirus carrying FAK shRNA. Reduced expressions of FAK, FAK-pY397, and paxillin-pY118 were observed in the irradiated cells, FAK shRNA-infected cells, and cells treated with combined treatment compared to control shRNA-infected cells (Figure 2A). Moreover, a diminished trend was more prominent in the FAK shRNA combined with irradiation treatment group. Additionally, the proportion of apoptotic cells was up to 7.86, 13.51, and 20.13% in the FAK shRNA, irradiation, and FAK shRNA combined with irradiation groups, respectively, compared to the control shRNA group (1.11%) (Figure 2B and Supplementary Figure 3).

To validate the long-term effect of cell growth, colony formation was evaluated in CAL27 cells exposed to carbon ions in the presence or absence of FAK. Compared to the irradiation alone treatment, the relative number of colonies formed by CAL27 cells after treatment with irradiation plus FAK knockdown was reduced by 47.2% (Figures 2C,D).

### Alterations in Cellular Motility Abilities Induced by FAK Silencing and Carbon Ion Irradiation

**Figure 3:**
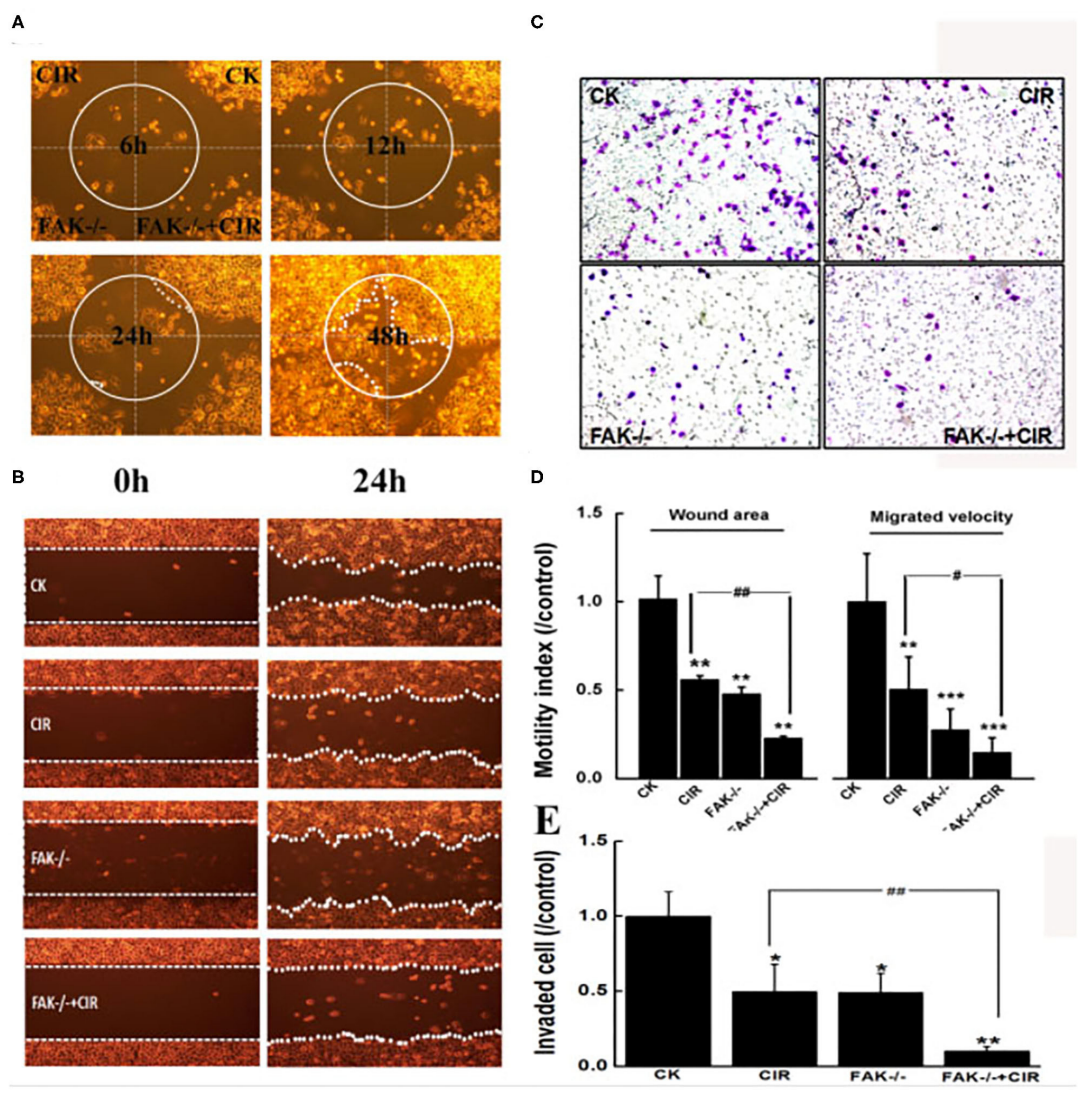
Inhibition of FAK reduces migration and invasion in CAL27 cells after carbon ion irradiation. **(A)** Monitoring of moving paths of the circular wound of cells up to 48 h. **(B)** Typical images of cells taken at 0 h and 24 h after scratching. **(C)** Representative images of cells invaded through the matrigel-coated membrane. **(D)** Quantitative analyses of cell migration signatures. **(E)** Quantitative evaluation of cell invasion ability. *P < 0.05, **P < 0.01, and ***P < 0.001 compared to the control group. ^#^*P* < 0.05 and ^##^*P* < 0.01 vs. the irradiation group.

The motility abilities of CAL27 cells following FAK deletion before carbon ion irradiation were evaluated via wound healing and Transwell invasion assays. The moving paths of circular wound recovery showed a decreased migration velocity of collective cells within 48 h in every treatment group compared to that in the shRNA-control group (Figure 3A). Moreover, the quantitative results of cells taken at 24 h after scratching indicated a markedly significant decline in the wound area by 44.6, 52.4, and 77.2% in irradiated, FAK-/-, and FAK-/- irradiated cells compared to that in control cells (Figure 3D). Further data revealed that a reduction in wound healing ability was significantly found in the FAK silencing and irradiation groups compared to the irradiation group (*P* < 0.01).

The Transwell invasion assay showed that the invasiveness capacity of CAL27 cells in irradiated cells (*P* < 0.05), FAK shRNA-infected cells (*P* < 0.05), and cells treated with FAK shRNA combined with irradiation (*P* < 0.01) was remarkably lower than that of control cells (Figure 3C). The number of invaded cells subjected to irradiation alone was 4.83-fold higher than that of cells treated with the combination of FAK shRNA infection and carbon ion irradiation (Figure 3E).

### Modulation of Cytoskeletal Rearrangement and Biomechanical Properties via FAK Downregulation

**Figure 4:**
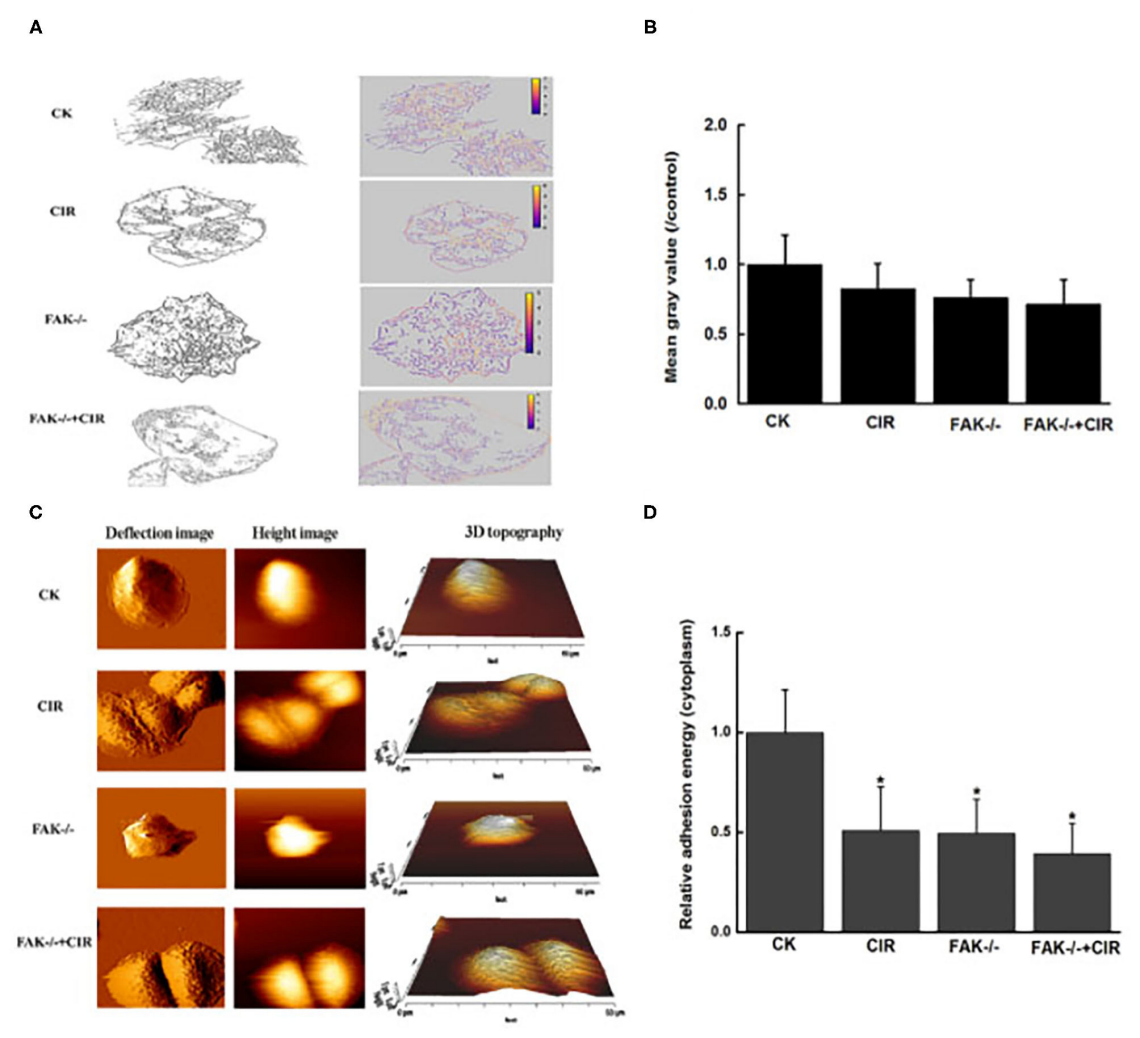
Modulation of cytoskeletal structure and cellular adhesion after treatment with carbon ion irradiation in the presence or absence of FAK activity. **(A)** Characteristic extraction of cytoskeletal structure. **(B)** Measurement of the relative mean gray value of cells in images. **(C)** Representative examples of vertical deflection, height, and 3D images in a single cell. **(D)** Quantitative analysis of adhesion energy in the cytoplasmatic region. The mean ± SEM (*N* = 3) were calculated for each value. *P < 0.05 vs. the control group.

As shown in Figure 4A and immunofluorescence staining in Supplementary Figure 2, an unordered actin filament arrangement accompanied by decreased intensity of actin fibers was observed in CAL27 cells with different treatments. In particular, actin staining exhibited an obvious ring-like distribution around the membrane protrusion structures of cells in the combination treatment group with FAK inhibition and irradiation. Moreover, in contrast to the control cells, FAK inhibition remarkably disrupted the formation of lamellipodia, filopodia, and membrane protrusions in the irradiated cells. Moreover, the abundance of the extracted cytoskeleton decreased in all treatment groups compared to that in the control group, but this trend was not significant.

The topography and deflection images are displayed in Figure 4C. Compared to the control group, there was a relatively rough plasma membrane in the combined treatment group. At this point, the variations in cytoplasmic adhesion energy from force-displacement curves acquired by the AFM indentations were decreased in the irradiated cells, FAK shRNA-infected cells, and cells treated with FAK shRNA combined with irradiation compared to the control cells (Figure 4D).

## Discussion

More aggression is found in TSCC than in other forms of OSCC because of its propensity for rapid local invasion and spread (31). As shown in Supplementary Figure 1, from the TCGA database, FAK/PTK2 was identified as one of the valuable diagnostic biomarkers in OSCC. Our data from nine patients with stage I TSCC (N0) and 47 patients with stage IV TSCC (≥N1) using public databases showed that genomic alteration of FAK closely modulated the malignant progression of TSSC, including histological differentiation, TNM stage, and lymph node metastasis (Figure 1). FAK is a critical non-receptor tyrosine kinase involved in many aspects of the metastatic process, including adhesion, migration, and invasion (32). Therefore, FAK could be a more suitable candidate for predicting the metastatic status of TSSCs than other existing biomarkers. Here, we propose that targeting FAK may present a feasible approach for improving the efficacy of radiotherapy for TSCC.

In previous studies, carbon ion irradiation as a promising therapy has been proven to efficiently induce cell death in X-ray-resistant OSCC by modulating diverse signaling molecules, such as AKT and SPHK1 (33, 34). Analogously, FAK has been reported to exert an anti-apoptotic action against ionizing radiation in HL-60 cells by inhibiting the mitochondrial apoptosis pathway (35). Our data showed that carbon ion irradiation remarkably repressed the expression of FAK and phosphorylation of FAK on Tyr^397^ and paxillin on Tyr^118^ in CAL27 cells. Furthermore, when cells were exposed to the combination of FAK downregulation by shRNA and irradiation, the clonogenic formation assay revealed a strong inhibitory effect on cell survival, along with a higher proportion of apoptotic cells (Figure 2). In agreement with previous findings, glioblastoma (36), colon cancer (37), and head and neck cancer cells (38) showed enhanced cell-killing effects in response to ionizing radiation by FAK deletion.

The activation of the FAK-paxillin signaling pathway has been considered a crucial index for tumor metastasis (39). In the current study, carbon ions distinctly limited the area and speed of wound healing cell migration as well as the number of invading cells through the invasion chamber (Figure 3). It is worth noting that, after FAK knockdown, the migratory and invasive abilities of CAL27 cells were more prominently inhibited when compared to cells treated with radiation alone. Here, the precise role of the combination of FAK knockdown and carbon ion irradiation was confirmed to suppress the high-motility capability of the TSCC cell line CAL27. Moreover, our findings suggest that FAK activity helps to elucidate the molecular mechanisms underlying motility reduction caused by carbon ion beams in TSCC. In this regard, previous findings have also demonstrated that heavy ion radiotherapy is more effective than conventional photon beam therapy in preventing metastasis in preclinical studies (40).

To provide further evidence for FAK knockdown-mediated cell motility repression, alterations in cytoskeletal structure and cell adhesion were determined in irradiated CAL27 cells. Cell movement relies on changes in the dynamics of actin filaments (41). Figure 4 shows that a certain decrease in the mean actin density was observed in each treatment group. However, as expected, as shown in Supplementary Figure 2, obvious disorganization of actin distribution and reduction of cell protrusion occurred in the combined FAK shRNA plus irradiation group, indicating that there is a lack of cell polarization and can limit cell motility potential. Furthermore, compared to control cells, FAK silencing resulted in reduced adhesion energy to the substrates in the irradiated cells, FAK shRNA-infected cells, or cells treated with FAK shRNA combined with irradiation, which was consistent with changes in cell motility and phosphorylation of adhesion proteins.

This study demonstrated that carbon ion irradiation and FAK inhibition, especially their combined application, can effectively inhibit OSCC, providing a basis for further *in vivo* experiments and clinical trials.

In addition, the detection of cytoskeletal structure and cell adhesion illustrated the relationship between their variation and cell motor ability. In future studies, we will further study the effects of heavy ions and FAK on cell properties, such as shape, elasticity, rigidity, and viscosity, as well as the relationship between cell properties and their migration and invasion ability, to find a more appropriate way to inhibit the metastasis of OSCC.

## Conclusion

Mechanistically, we have provided evidence that carbon ion irradiation significantly blocked the FAK-related signal pathway, which partially explains the anti-tumor mechanisms of carbon ions. Furthermore, combining FAK downregulation with carbon ion irradiation could synergistically offer comparable therapeutic benefits for TSCC patients regarding the inhibition of metastatic potential.

## Data Availability Statement

The raw data supporting the conclusions of this article will be made available by the authors, without undue reservation.

## Ethics Statement

The studies involving human participants were reviewed and approved by Tissue microarray chip containing oral squamous cell carcinoma were obtained from Shanghai Biochip Co. The ID of ethics approval is T20-0361, submitted in this system. The patients/participants provided their written informed consent to participate in this study.

## Author Contributions

QS: designed the experiment, executed the research and wrote the original draft. QY, ZB, RF, and XH: executed the research and interpreted the data. BL and JW: discussed reviewed the manuscript. XA and YL: acquired the data, analyzed the data and interpreted the data, and reviewed the manuscript. All authors contributed to the article and approved the submitted version.

## Conflict of Interest

The authors declare that the research was conducted in the absence of any commercial or financial relationships that could be construed as a potential conflict of interest.

## Abbreviations

AFM: Atomic Force Microscope
CK: control check
CIR: carbon ion radiotherapy
FAK: focal adhesion kinase
JCT: juxtacancerous tissue
OSCC: oral squamous cell carcinoma
PORT: postoperative radiation therapy
PTK2: protein tyrosine kinase 2
TSCC: tongue squamous cell carcinoma.
