# Supplementary material for: Carbon Ion Irradiation Enhances the Anti-tumor Efficiency in Tongue Squamous Cell Carcinoma via Modulating the FAK Signaling
Source: Front Public Health. 2021 Feb 3;9:631118. doi: 10.3389/fpubh.2021.631118 (PMC7901966; doi:10.3389/fpubh.2021.631118)
Supplement: Supplementary file 1 [file Table_1.DOCX]

Supplementary Material

## Supplementary Figures


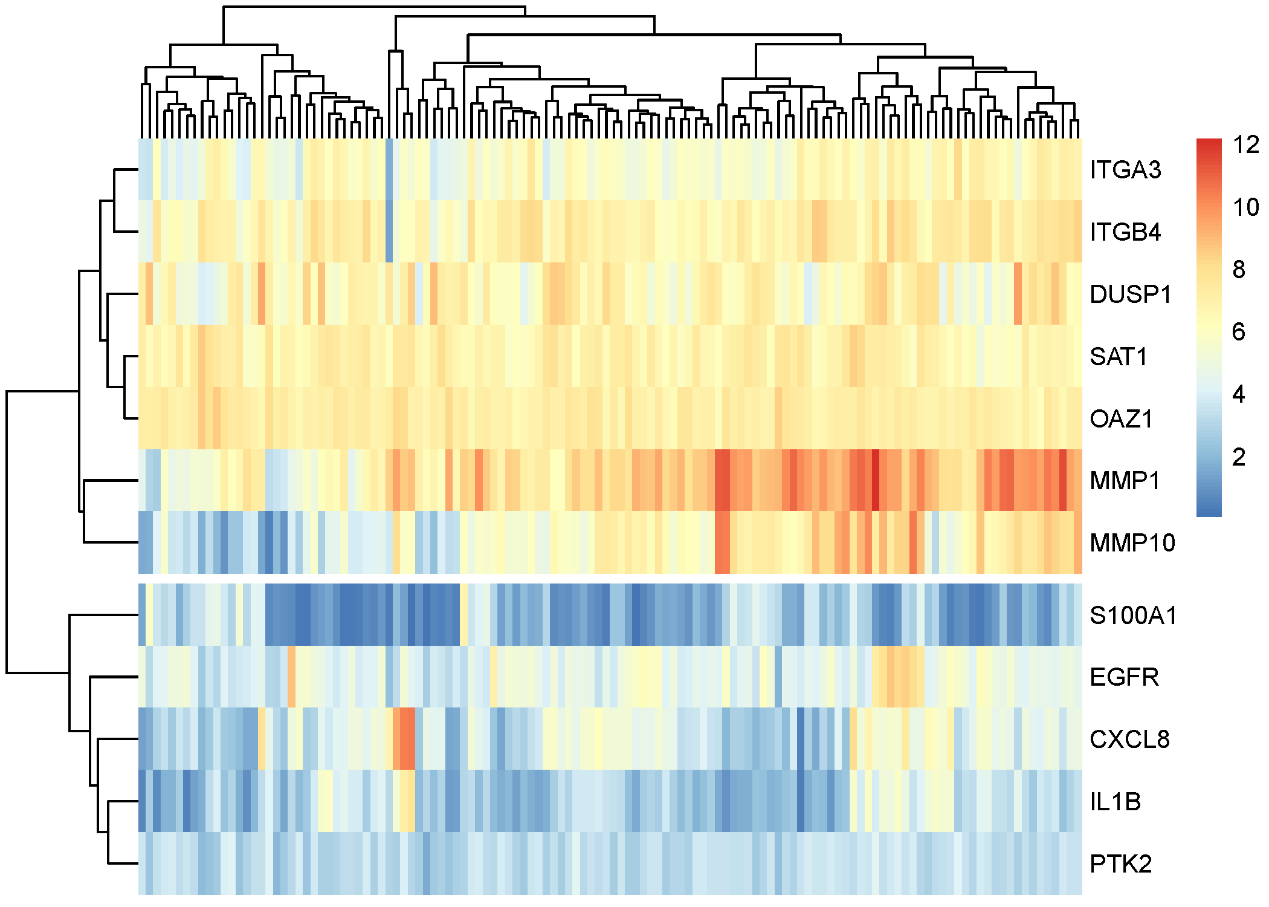


**Supplementary Figure 1.** Differential gene expressions for Oral squamous cell carcinoma as valuable diagnostic biomarkers from The Cancer Genome Atlas (TCGA) database.


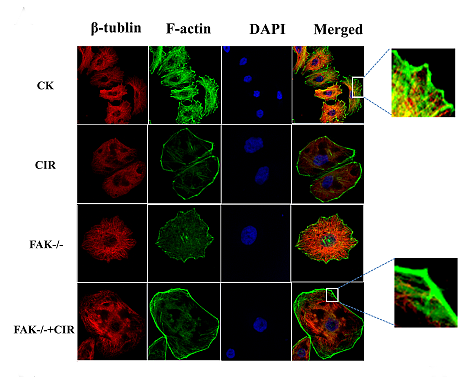


**Supplementary Figure 2.** Classical photos of the cytoskeletal array and amplified membrane protrusions. Cells were stained with AlexaFluor647-labeled anti-β-tubulin (Red). F-actin staining was detected with fluorescein isothiocyanate- phalloidin (Green).


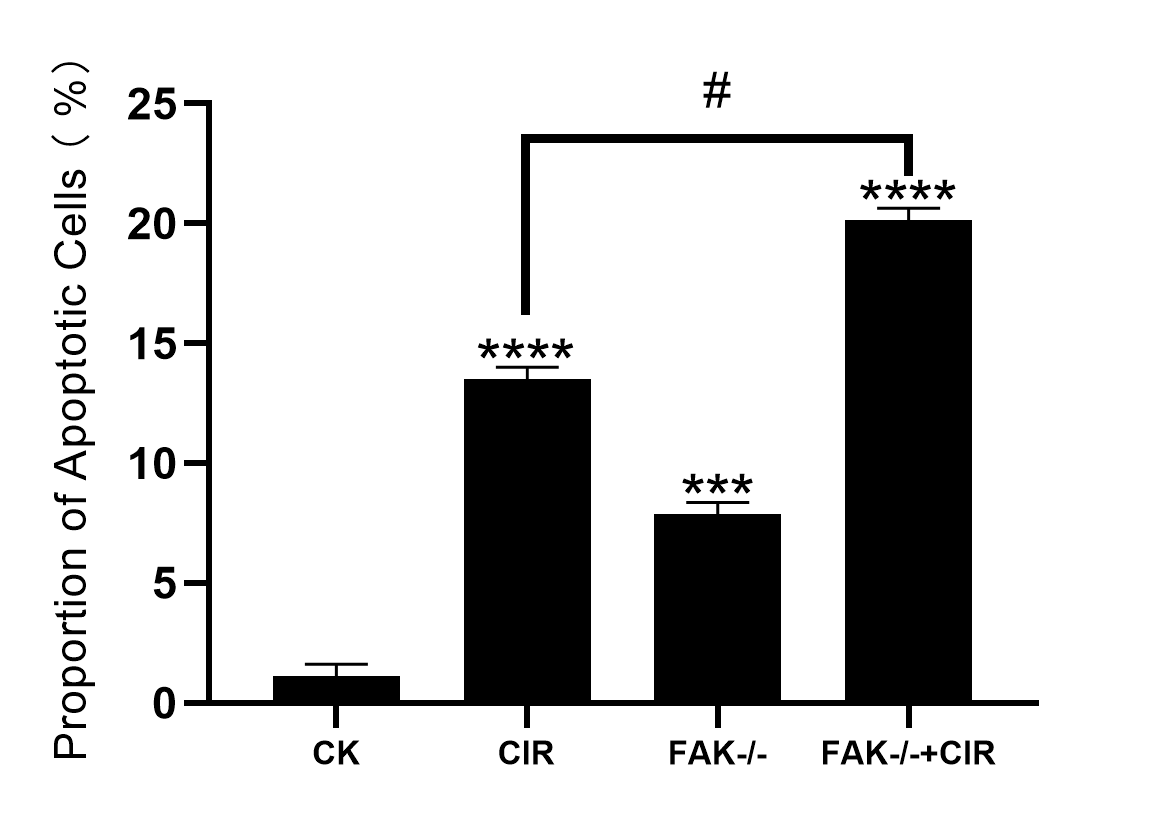


**Supplementary Figure 3.** Quantitative evaluation of the proportion of apoptotic cells by flow cytometry. The means ± SEM (N = 3) were calculated for each value. ***P < 0.001 vs. the control group. ****P < 0.0001 vs. the control group. #P < 0.0001 vs. the irradiation group.
